# Maternal administration of anti-angiogenic agents, TNP-470 and Angiostatin_4.5_, induces fetal microphthalmia

**Published:** 2009-06-26

**Authors:** Catrin S. Rutland, Keyi Jiang, Gerald A. Soff, Christopher A. Mitchell

**Affiliations:** 1 Academic Division of Obstetrics and Gynaecology, Nottingham City Hospital, University of Nottingham, Nottingham, UK; 2 Feinberg Medical School of Northwestern University, Chicago, IL; 3 Centre for Molecular Biosciences, University of Ulster at Coleraine, Coleraine, Co. Londonderry, UK

## Abstract

**Purpose:**

Agents specifically targeting the vasculature as a mode of therapy are finding increasing use in the clinic, primarily in the treatment of colon cancer (Avastin™) and age-related macular degeneration (Lucentis™). We have previously shown that maternal administration of angiogenic inhibitors (TNP-470 [O-[chloroacetyl-carbamoyl]fumagillol, initially called AGM-1470], the first angiogenic inhibitor to undergo clinical trials, and Angiostatin_ 4.5_, currently in phase I-III clinical trials) cause fetal growth restriction and/or placental abnormalities. During a rapid growth phase of ocular development in the mouse (embryonic days 12 to 19 [E12-E19]), the placenta mediates the metabolic requirements of the fetus and consequently may impact upon the growth of the highly oxygen sensitive fetal eye.

**Methods:**

We injected pregnant dams (between E10.5 – E18.5) with anti-angiogenic agents, which caused either a placental insufficiency type of IUGR (intrauterine growth restriction; i.e., TNP-470) or frank placental pathology (Angiostatin_4.5_ [AS_4.5_]), and assessed changes in absolute ocular dimensions, tissue types, and vascular profiles using stereological techniques.

**Results:**

The experiments showed that ocular volumes were significantly reduced in fetal mice where dams were treated with either TNP-470 or AS_4.5_. Furthermore, TNP-470 specifically caused a reduction in hyaloid blood vessel length and volume, the only intraocular vascular circulation in fetal mice.

**Conclusions:**

These experiments support the hypothesis that the angiogenic inhibitors (specifically TNP-470 and AS_4.5_) induce microphthalmia either indirectly by their known effects on placental morphology (and/or function) or directly via altering microvascular growth in the fetus. These results also warrant further investigation of a new experimental paradigm linking placental pathology-related fetal growth restriction and microphthalmia.

## Introduction

Angiogenesis inhibitors are now finding widespread clinical use as first-line treatments for ocular conditions such as age-related macular degeneration (e.g. Lucentis™) or as adjuvant chemotherapeutic agents in the management of colorectal carcinoma (e.g., Avastin™) in addition to being assessed for efficacy in a large variety of clinical trials for specific neoplasms [1,2]. Although there are large numbers of clinical trials with these agents, there is relatively little information regarding their effect on fetal growth and development despite pregnancy being a contraindication for entry into many of these trials. Since the worldwide use of these agents is likely to increase dramatically in the coming years, information on their potential teratogenic effects particularly in rapidly growing fetal organs or tissues having a high metabolic rate (such as the eye) will be important to increase the knowledge base on this mechanistically diverse range of compounds. TNP-470 (O-[chloroacetyl-carbamoyl]fumagillol, initially called AGM-1470) is a semi-synthetic derivative of fumagillin, a naturally secreted antibiotic of *Aspergillus fumigatus* fresenius [3]. The target of TNP-470 was identified as the type 2 methionine aminopeptidase (MetAP2) [4]. One important role of the methionine aminopeptidases is the posttranslational processing required for protein myristoylation [4]. Further studies showed that TNP-470 blocks S-phase entry and that this cell cycle blockage is characterized by the hypophosphorylation of the retinoblastoma protein (pRB), which is likely due to the dramatic inhibition of cyclin E-dependent kinase activity [5]. It was also demonstrated that the inhibition of cyclin-dependent kinase (CDK) activity is caused by the upregulation of the CDK inhibitor, p21^WAF1/CIP1^ (p21), which in turn is activated by p53 [5]. Angiostatins consist of varying numbers of the kringle domains [1-5] of plasminogen with Angiostatin_4.5_ (AS_4.5_; the subject of this study) [6] being a naturally occurring cryptic fragment consisting of kringles 1–4 and most of kringle 5. Angiostatin is produced by at least two distinct mechanisms: first, via the binding of plasminogen to the cell membrane by β-actin and uPAR followed by proteolytic cleavage by urokinase-like plasminogen activator/tissue plasminogen activator and autoproteolysis [7] and second, via the proteolytic cleavage of plasminogen through neutrophil elastase, which is produced by activated human neutrophils [8]. Liberation of angiostatin by circulating neutrophils results in increased expression of Interleukin-12 in macrophages [9], implicating the innate immune system in its anti-angiogenic activities. Angiostatin also binds to one uncharacterized binding protein (ABSP) [10] and angiogenin [11] (a potent inducer of angiogenesis), the latter of which may play a role in mediating the well documented anti-angiogenic effects of angiostatin. Angiostatin 4.5 has also been shown to induce endothelial cell apoptosis [12,13] by activating a caspase cascade, specifically the activation of Caspases 3, 8 and 9 [12]. Angiostatin binds to cell surface proteins (annexin II [14], the chondroitin sulfate proteoglycan NG2 [15], c-met [16], angiomotin [17], ATP synthase [18], and α_v_β_3_ integrin [19]), which have been shown to mediate its pleiotropic actions including inhibition of endothelial migration, filopodial extension, tube formation, and apoptosis in vitro as well as tumor growth in vivo. Blocking these receptors with monoclonal antibodies or soluble proteins inhibits both the binding of angiostatin as well as its activity in vitro and in vivo assays. It is clear that angiostatin (like its parent molecule, plasminogen) is promiscuous in its binding partners and that the functional activities of this potent anti-angiogenic protein are consequently mediated through a variety of molecular pathways [20].

During early murine pregnancy, a maternal injection of TNP-470 (the first angiogenesis inhibitor to be assessed in clinical trials) results in spontaneous abortion [21] whereas administration during the second half of murine pregnancy results in a reproducible model of intrauterine growth restriction (IUGR) in mice [22]. Furthermore, Angiostatin_4.5_ (AS_4.5_) induces placental abnormalities in addition to fetal growth abnormalities following maternal administration in the second half of murine pregnancy [23]. Findings with these two angiogenic inhibitors are consistent with the concept that angiogenic inhibitors directly affect placental development and fetal growth. The placenta, which is the materno-fetal interface that mediates the metabolic requirements of the fetus, allows the ready passage of a variety of molecules with low molecular weights (M_w_: up to 1 kDa) whereas large proteins (such as heparin and insulin) do not cross this barrier unless there is an active transport mechanism [24]. In addition to having direct effects on the placenta, small M_w_ angiogenic inhibitors such as thalidomide also have direct teratogenic effects on the fetus [25] at least partially via their effects on the vasculature [26]. Considering that many of the angiogenic inhibitors are low molecular weight compounds [27-29] and are thus likely to cross the placenta or have adverse effects on placental growth, many of these molecules may also affect fetal growth in general, including ocular development.

The developing eye is extremely sensitive to alterations in oxygen concentrations [30-34] and vascular morphological changes mediated by altered expression of vascular endothelial growth factor (VEGF) [35,36]. Since the oxygen concentrations sensed by the fetal eye are ultimately mediated via placental transport, agents that influence placental vascular growth such as TNP-470 and AS_4.5_ are highly likely to influence ocular development. To test this hypothesis, we administered angiogenic inhibitors with two differing modes of action (i.e., TNP-470 and AS_4.5_) to pregnant dams. We then used stereological analysis to determine their effect on fetal ocular dimensions and on the volumes of the individual tissue types including the hyaloid vasculature (HV), which supports the early growth of the fetal eye.

The experiments in this paper were designed to assess the effects of maternal administration of either TNP-470 or AS_4.5_ on overall ocular morphology and particularly the vascular compartment of the fetal eye. TNP-470 is a low molecular weight angiogenesis inhibitor (M_w_=401.89), known to induce murine IUGR, and characterized by significant placental morphological changes. AS_4.5_ (M_w_ ~52–55 kDa) [7] also induces placental pathology without IUGR, although there is notable fetal skeletal growth delay [23], which is suggestive of a placental insufficiency. The null hypotheses for these studies are that ocular morphology and dimensions in addition to the cross-sectional area or volume of hyaloid blood vessels are not significantly altered in the eyes of fetuses from dams treated with angiogenic inhibitors in comparison with vehicle-only treated control mice.

## Methods

### Animal model

The experiments reported in this study were performed following appropriate local and national (Home Office) ethical approval, which are equivalent to the Association for Research in Vision and Ophthalmology (ARVO) guidelines and the Institute for Laboratory Animal Research (Guide for the Care and Use of Laboratory Animals) guidelines. Adult male and female C57BL/6J mice (eight weeks old) were housed in a 12-h on/12-h off light-dark schedule. After mating, the presence of a vaginal plug was defined as embryonic day 0.5 (E0.5). Pregnant dams were randomly assigned to a group, which received either PBS or 30 mg/kg bodyweight TNP-470 (n=19 and n=17, respectively). Subcutaneous injections of TNP-470 were administered every other day from E10.5 to E18.5. Alternatively, pregnant dams were randomly assigned to a group receiving 20mg/kg bodyweight AS_4.5_ (n=6) daily from E10.5-E18.5. Dose and injection times and efficacies were in line with previous studies [21,37-40]. The biological activity of both TNP-470 and AS_4.5_ was confirmed in vitro before testing in vivo [12,22].

Following euthanasia, the gravid uteri were carefully dissected free from the mother. After amnionectomy, the fetuses were delivered and euthanized, and the eyes were enucleated. One eye from each embryo was fixed in 10% buffered formal saline (BFS; pH 7.4) overnight and subsequently embedded in araldite.

### Ocular stereology

A total of 10 vehicle-only controls, 6 TNP-470-treated mice, and 5 AS_4.5_-treated mice were randomly selected from the total population of collected fetuses. Following fixation, eyes were processed, critically orientated in a mold, and embedded in araldite. Serial sections (0.5 μm thick) were cut at 50 μm intervals through the eye. The sections were placed onto glass slides and subsequently stained with 2% toluidine blue. A three-stage stereological analysis was performed to determine (i) ocular volume, (ii) tissue and vascular proportions, and (iii) vascular morphometry using systematic random sampling [41,42]. Light microscopic images were obtained using an Olympus microscope (Olympus, Tokyo, Japan) and electronic images were captured with an Olympus T4040 digital camera. Each section was visualized, and stereological analysis was performed using the ‘QProdit’ computer imaging program (Leica Imaging Systems, Cambridge, UK).

#### Ocular volume

The perimeter of each eye section was traced and the area calculated. Cross-sectional areas from individual sections were multiplied by 50 (to take into account that sections were cut at 50 µm intervals) and subsequently summed to determine fetal ocular volume.

#### Tissue proportions

Serial sections (0.5 μm thick) of each eye were cut at 50 μm intervals through the eye, and two systematically random views [42] of each eye section were collected, stored, and analyzed with the aid of a 96-point grid layout (this resulted in an average of 50 sections being analyzed per eye, ~4,800 points per eye). In late fetal mice, there are two distinct anatomic regions of the hyaloid vasculature (HV), one surrounding the lens (tunica vasculosa lentis [TVL] incorporating the pupillary membrane anteriorly) and the other on the vitreal surface of the inner limiting membrane (arteria hyaloidea propria; AHP) [43]. With respect to the stereological analyses of ocular tissue dimensions, the AHP and TVL were considered separately. The ocular tissues were thus assigned to 1 of 11 tissue type groups based on their location and histological phenotype: retina, lens stroma, cornea, vitreous humor, aqueous humor, iris, lens hemorrhage, TVL, AHP, optic stalk, and non-ocular tissue (includes the sclera and eyelid). Tissue proportion refers to the relative proportion of each tissue/blood vessel type within the whole eye.

#### Vascular morphometry

A photomicrograph of each section (averaging 25 sections per eye) containing HV was analyzed by tracing around each blood vessel (an average of 70 tracings per eye). Vessels within the ‘broken lines’ were included within the count whereas vessels crossing the ‘solid lines’ were excluded. Blood vessel lengths, cross-sectional areas, diameters, and volumes were calculated using the tissue proportion and ocular volume data [44].

### Statistical analysis

Statistical comparisons between groups were assessed using Levene’s test for equality of variances. Following confirmation of homologous populations, a *t*-test for equality of means could be used with a p<0.05 being considered significant.

## Results

### The effect of angiogenic inhibitors, TNP-470 and AS_4.5_, on murine pregnancy

The effect of angiogenic inhibitors on placental and fetal development were similar to those described previously by our group for TNP-470 [22] and AS_4.5 _[23] (unpublished). In brief, murine dams injected with 30 mg/kg of TNP-470 showed consistent weight loss in the latter half of pregnancy with reduced placental size and altered ratios of fetal to maternal vessels and fetal growth restriction. Dams injected with AS_4.5_ consistently gained weight during pregnancy, and the placental weight and dimensions were normal as were litter sizes and fetal weight. Placentae from AS_4.5_-treated dams had significant pathological changes, consistent with intravascular coagulation and vascular restructuring (data not shown). Fetuses from AS_4.5_-treated dams also had marked signs of skeletal growth delay and widespread edema [23] (data not shown).

### Stereological analysis

#### Ocular volume

**Figure 1:**
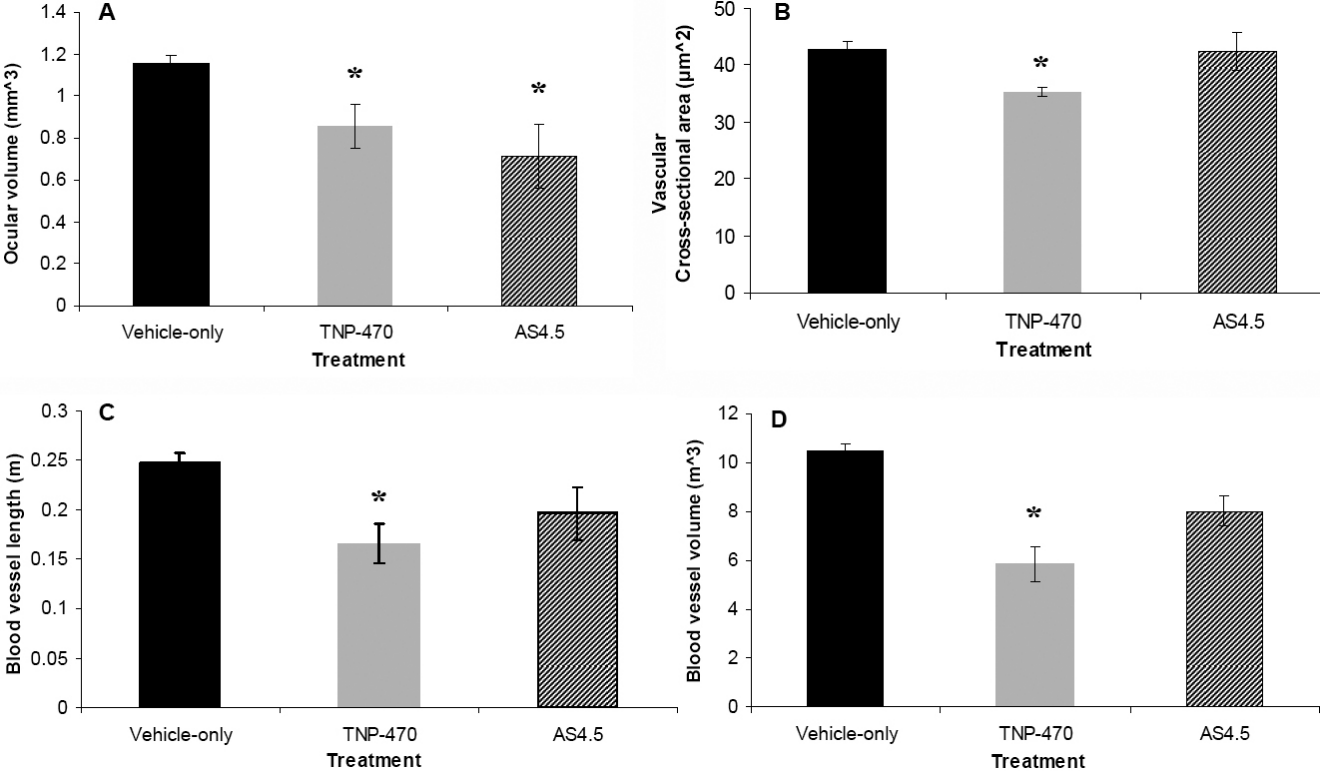
Hyaloid blood vessel dimensions from E18.5 mice treated with vehicle-only, TNP-470, or AS_4.5_ solution. The graphs show ocular volume (**A**) where the asterisk indicates p<0.04, vascular cross sectional area (**B**) where the asterisk indicates p<0.01, vascular length (**C**) where the asterisk indicates p<0.001, and vascular volume (**D**) where the asterisk indicates p<0.001 in vehicle-only (control), TNP-470-treated, and AS_4.5_-treated animals. Statistical comparisons between groups were performed using *t*-test for equality of means.

**Figure 2:**
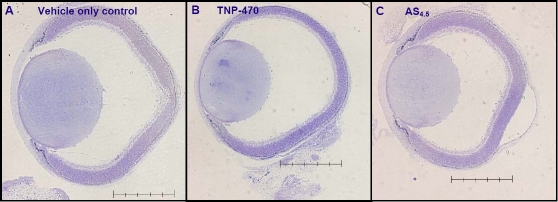
Photomicrographs of the eye and associated structures from E18.5 mice treated with vehicle-only, TNP-470, or AS_4.5_. Photomicrographs of representative E18.5 murine eyes stained with toluidine blue are as follows: vehicle-only treated (**A**), TNP-470-treated eyes (**B**), and AS_4.5_-treated eyes (**C**). The ocular cross-sectional area is clearly smaller in the TNP-470- and AS_4.5_-treated groups when compared with controls (compare with Figure 1). In the images, the scale bar=500 μm.

Maternal administration of TNP-470 resulted in a 27% reduction in ocular volume in comparison with vehicle-only treated eyes (p<0.04; Figure 1A and Figure 2). Administration of AS_4.5_ led to a 38% reduction in ocular volume in comparison to vehicle-only treated eyes (p<0.04; Figure 1A and Figure 2).

#### Tissue proportion stereology

TNP-470-treated fetal eyes showed a 40% reduction in the proportion of TVL (p=0.04) and a 59% increase in the iris (p=0.02) when compared with controls. Tissue proportions of all other tissues were not significantly different. In fetal eyes from dams exposed to AS_4.5_, there was a 61% reduction in the size of the optic stalk (p=0.003; see Table 1) with no other significant differences in tissue proportions observed.

**Table 1:** Proportions of ocular tissue types following maternal administration of vehicle only, TNP-470, or AS_4.5_ solution.

**Tissue**	**Tissue label**	**Vehicle-only (n=10)**	**30 mg TNP-470 (n=6)**	**p**	**20 mg AS_4.5 _(n=5)**	**p**
Retina	A	0.3681±0.0141	0.3211±0.0471		0.3724±0.0330	
Cornea	B	0.0340±0.0034	0.0303±0.0079		0.0231±0.0053	
Optic Stalk	C	0.0062±0.0006	0.0049±0.0016		0.0024±0.0007	0.003
Aqueous	D	0.0027±0.0001	0.0022±0.0007		0.0043±0.0003	
Vitreous	E	0.3519±0.0073	0.3939±0.0986		0.3702±0.0573	
TVL	F	0.0035±0.0004	0.0021±0.0004	0.04	0.0026±0.0007	
AHP	G	0.0057±0.0009	0.0050±0.0009		0.0062±0.0007	
Iris	H	0.0232±0.0027	0.0368±0.0039	0.02	0.0231±0.0048	
Lens stroma	I	0.1923±0.0954	0.2037±0.0056		0.1956±0.0143	
Total HV	F+G	0.0093±0.0011	0.0071±0.0009		0.0088±0.0012	

Values represent mean±SEM. Statistical comparisons between groups were performed using *t*-test for equality of means. Significance was accepted as p<0.05. The p value is only indicated where a significant difference was observed between the vehicle-only (control) and experimental groups.

#### Ocular blood vessel stereology

**Figure 3:**
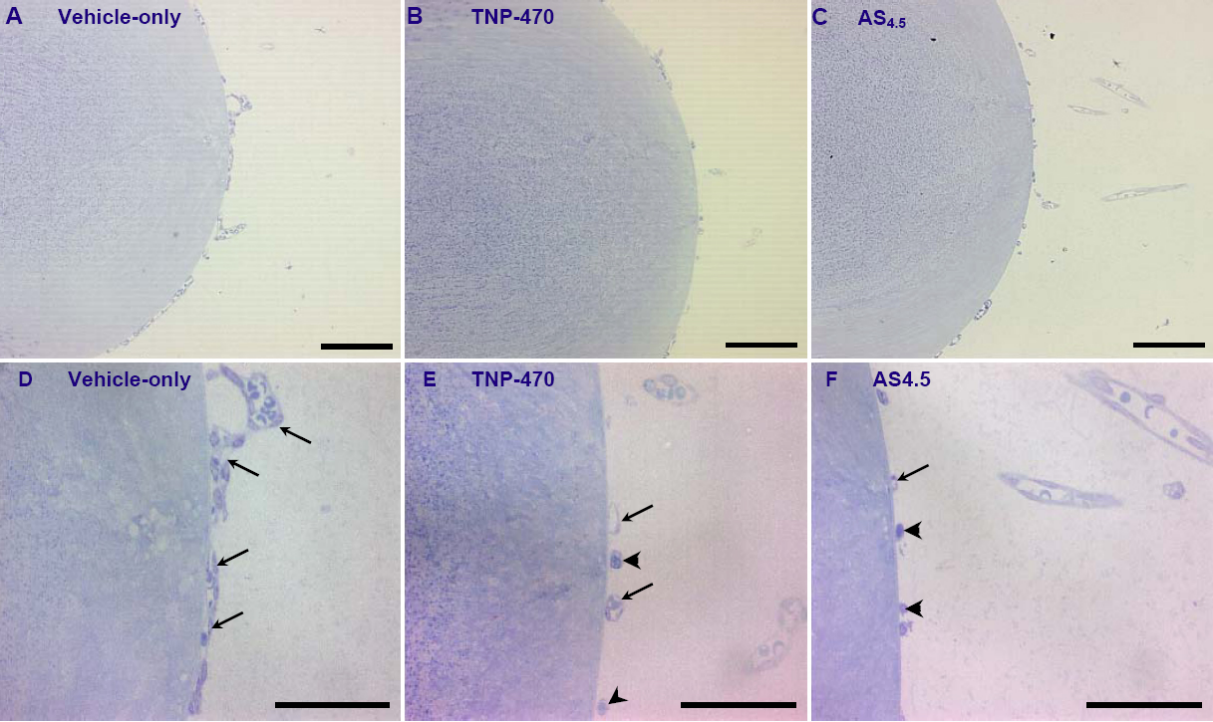
Photomicrographs of the lens and hyaloid vasculature in eyes from E18.5 mice treated with either vehicle-only, TNP-470, or AS_4.5_ solution. Low power micrographs (**A**-**C**) and matching high power detail (**D**-**F**) of the lens and hyaloid vasculature in eyes from E18.5 mice stained with toluidine blue are displayed. Dams were treated with either vehicle-only (control: **A**,**D**), TNP-470 (**B**,**E**), or AS_4.5_ (**C**,**F**)_._ In high power light micrographs (**D**-**F**), the hyaloid vessels are clearly visible on the lens surface (arrows) in addition to hyalocytes (arrowheads). Scale bars on **A**-**C**=100 µm and on **D**-**F**=50 µm.

Treatment with TNP-470 resulted in a decrease in length of the HV by 36% in fetal mice (p<0.01; Figure 1C and Figure 3B)  and cross-sectional areas (p<0.001; Figure 1B and Figure 3B). HV volumes were nearly halved in TNP-470-treated fetal eyes in comparison with control mice (p<0.001; Figure 1D and Figure 3). All other comparisons were not significantly different (p>0.05; Figure 1 and Figure 3).

## Discussion

Anti-angiogenic therapies for treatment of either solid tumors or non-neoplastic conditions generally exhibit low levels of toxicity because they target the vascular compartment allowing lower dosages to be used [45]. These agents have seen a rapid increase in clinical usage since their approval by the NIH in 2002 for treatment of colon cancer and ocular conditions characterized by aberrant vascular formation (notably “wet” age-related macular degeneration). Therefore, possible teratogenic effects warrant further investigation. In the present study, fetuses exposed to either the small molecular weight endothelial inhibitor TNP-470 (Mw ~402 Da) or AS_4.5_ (52–55 kDa) showed a significant decrease in fetal ocular volume, which may be associated with fetal growth restriction (FGR) and placental pathologies resulting from maternal administration of these agents [22]. Microphthalmia is observed in conjunction with FGR in several other clinically relevant disorders including those affected by Matthew-Wood syndrome [46] and Fanconi anemia [47], patients with deletions in 3q26.33-q28 [48], and those with the X-linked microphthalmia with linear skin defects syndrome [49]. Fetal growth restriction and microphthalmia have also been observed in rats exposed to antiserum targeting the visceral yolk sac endoderm [50] or hyperthermia [51]. Microphthalmia is also frequently observed in children with fetal alcohol syndrome [52-54] with reduced globe size and weight being observed in pre- and post-natal rodents chronically exposed to ethanol [55,56]. In addition to microphthalmia, ocular effects such as a reduction in retinal thickness [57], persistent hyperplastic primary vitreous (PHPV), and smaller lens vesicles [58,59] have been observed in ethanol-exposed animals, although none of these pathologies were observed in the present study. While abnormalities in the developing iris such as coloboma are observed in ethanol-treated mice [52], no distinct pathology other than an increase in iridial size was observed in TNP-470-treated mice.

In humans, HV has completely regressed by the seventh month of gestation, although in rodents the vessels persist until the third to fourth week post-natally [60]. Inappropriate neovascularization within the human eye contributes to visual loss in several ocular diseases including retinopathy of prematurity [61] and PHPV, which can have several different ocular manifestations ranging from persistent pupillary membrane, Mittendorf dot, and even microphthalmia [62]. Examination of TVL is an accurate method for determining fetal age [63], especially in the case of infants small for their gestational age [64]. In infants whose weights are within or below the tenth percentile (lowest 10% weight group within a population), regression of TVL correlates well with their gestational age in agreement with infants of appropriate weight to gestational age [65]. In addition, the rate at which TVL regresses in prematurely delivered neonates is concordant with in utero infants, indicating that early delivery is not associated with accelerated regression of TVL [66]. Remnants of HV are often observed in human preterm infants (less than 38 weeks gestation), but the remnants regress in accordance to the length of time before term at which the infant was delivered [67].

Reduced blood vessel volume as a result of TNP-470 administration has been observed in several models of xenotransplanted human tumors, leading to clinical trials of this agent. A direct effect of TNP-470 on ocular vasculature has also been confirmed by a reduction in blood vessel length in over-vascularized corneas (caused by upregulation of VEGF) where vessel length was significantly decreased in TNP-470-treated animals [68]. Therefore, the results of the present experiment are consistent with the hypothesis that the low molecular weight angiogenic inhibitor, TNP-470, reduces HV length and volume in the murine eye. Due to the fast uptake, low molecular weight, and relatively long half-life of this compound [69], its effects are likely to be mediated through a direct effect on fetal vasculature.

VEGF expression from both the lens and retinal astrocytes is critical for developmental vascular growth in the hyaloid and retinal vascular plexi, respectively [35,44,70,71]. Several lines of evidence implicate VEGF signaling-mediated mechanisms of action for TNP-470. These include inhibition of VEGFR-2 phosphorylation and reduced Vascular Permeability Factor/VEGF-induced RhoA activation [72]. Administration of TNP-470 also causes a decrease in levels of VEGF in a variety of cell and tissue types [73-75] including the eye [68]. While angiostatin does not appear to directly influence VEGF signaling [76], it can modulate α_v_β_3_ integrin, which in turn influences VEGF expression [77]. Furthermore, in rat models of oxygen-induced retinopathy and streptozotocin-induced diabetes, angiostatin significantly reduces retinal vascular permeability and downregulates VEGF production while both permeability and VEGF levels remained unchanged in control animals [78]. As angiostatin binds to the α_v_β_3_ integrin and inhibits the p42/p44 mitogen-activated protein (MAP) kinase pathway, angiostatin-induced VEGF downregulation may be mediated via the inhibition of the MAP kinase pathway under conditions of hypoxic stress [78]. Taken together, these findings implicate the VEGF signaling pathway as the mechanism of action for these anti-angiogenic agents, although further investigation of this hypothesis is warranted.

Administration of AS_4.5_ has also been shown to cause a reduction in blood vessel volume in models of retinopathy [79], colonic anastomoses [80], and coronary angiogenesis in vivo [81]. In the present study, the proportion of capillaries (on either the inner limiting membrane or hyaloid vessels) was unaffected by the administration of AS_4.5_. However, optic nerve head hypoplasia was consistently observed in fetal mice exposed to 20 mg/kg of angiostatin in this study. Optic nerve head hypoplasia in association with reduced retinal vascularization is a well described clinical phenomenon [82], particularly in children delivered before 29 weeks of gestation [83] or with growth defects attributable to reduced levels of a growth hormone [84] or insulin-like growth factor 1 [85]. Since AS_4.5_ is a ~55 kDa protein, it is highly unlikely to cross the placenta where it induces significant placental pathology. Therefore, the effects on both ocular dimension and optic nerve head hypoplasia are likely to be mediated via the pathological changes observed in placentae of these mice (Rutland and Mitchell; unpublished observations). The clear association of optic nerve head hypoplasia with pre-term or low birth weight infants is consistent with reduced placental perfusion mediating this pathology and may explain this observation in the present study. In a published study involving intravitreal injection of angiostatin in neonatal mice [79], the progression of vascular malformations in an oxygen-induced retinopathy model was slowed without affecting the normal vasculature. Similarly, in a mouse model of proliferative retinopathy, stable expression of a human immunodeficiency virus vector-encoding angiostatin also inhibited retinal neovascularization by up to 90% [86], demonstrating that the anti-angiogenic effects of this protein are observed in vasculature adjacent to the injection site. Another interesting study investigated intravitreal injection of angiostatin in diabetic and control rats and measured vascular permeability [87]. The authors reported that pathological vascular permeability was reduced in the diabetic mice whereas permeability was unaffected in control mice [87].

Studies from our group and others have shown that angiogenic inhibitors are not entirely specific to pathologic angiogenesis [88] as TNP-470 clearly affects the physiologic angiogenesis associated with both early embryonic development and fetal-placental development [44]. In contrast, the effects of maternal administration of AS_4.5_ are consistent with placental-mediated effects on ocular dimensions as HV in fetal mice was unaffected. The small molecular weight compound, TNP-470 (Mw=402), can influence growth of a vasculature destined to regress before birth in humans (i.e., the hyaloid vascular system) whereas AS_4.5_ does not exert a direct effect on normal fetal vasculature via maternal administration. Intraocular injections of anti-angiogenic agents may prove useful in the treatment of early post-natal growth in disorders characterized by aberrant angiogenesis such as persistent hyperplastic primary vitreous. This study has shown that anti-angiogenic agents capable of inducing FGR can result in concomitant microphthalmia, providing evidence for contraindication of use of these agents during pregnancy.
